# The World Health Organization 2030 goals for *Taenia solium*: Insights and perspectives from transmission dynamics modelling: CystiTeam Group for Epidemiology and Modelling of *Taenia solium* Taeniasis/Cysticercosis

**DOI:** 10.12688/gatesopenres.13068.2

**Published:** 2019-12-13

**Authors:** CystiTeam Group for Epidemiology and Modelling of Taenia solium Taeniasis/Cysticercosis

**Keywords:** Taenia solium, taeniasis, (neuro)cysticercosis, mass drug administration, pig vaccination, cystiSim, EPICYST, One Health

## Abstract

*Taenia solium *(TS), responsible for porcine cysticercosis, human taeniasis and (neuro)cysticercosis, was included in the World Health Organization neglected tropical disease (NTD) roadmap published in 2012. Targets set in this roadmap have not been met, but *T. solium* has been included in the consultation process for the new 2030 goals proposed for priority NTDs. *Taenia solium* transmission dynamics models can contribute to this process. A recent review has compared existing *T. solium* transmission models, identifying their similarities and differences in structure, parameterization and modelled intervention approaches. While a formal model comparison to investigate the impact of interventions is yet to be conducted, the models agree on the importance of coverage for intervention effectiveness and on the fact that human- and pig-focused interventions can be optimally combined. One of these models, cystiSim, an individual-based, stochastic model has been used to assess field-applicable interventions, some currently under evaluation in on-going trials in Zambia. The EPICYST, population-based, deterministic model has highlighted, based on simulating a generic sub-Saharan Africa setting, the higher efficacy (measured as the percentage of human cysticercosis cases prevented) of biomedical interventions (human and pig treatment and pig vaccination) compared to improved husbandry, sanitation, and meat inspection. Important questions remain regarding which strategies and combinations thereof provide sustainable solutions for severely resource-constrained endemic settings. Defining realistic timeframes to achieve feasible targets, and establishing suitable measures of effectiveness for these targets that can be quantified with current monitoring and evaluation tools, are current major barriers to identifying validated strategies. *Taenia solium* transmission models can support setting achievable 2030 goals; however, the refinement of these models is first required. Incorporating socio-economic elements, improved understanding of underlying biological processes, and consideration of spatial dynamics are key knowledge gaps that need addressing to support model development.

## Abbreviations

CLTS, community-led total sanitation intervention; DALY, disability-adjusted life year; EOT, elimination of transmission; FOI, Force-of-infection; MDA, mass drug administration; M&E, monitoring and evaluation; NCC, neurocysticercosis; NTD, neglected tropical disease; PCC, porcine cysticercosis; PAHO, Pan American Health Organization; SAC, school-age children; SSA, sub-Saharan Africa; TS, *Taenia solium*; WHO, World Health Organization; zDALY, zoonotic disability-adjusted life year.

## Disclaimer

The views and opinions expressed in this article are those of the authors and do not necessarily reflect those of the World Health Organization. Publication in Gates Open Research does not imply endorsement by the Gates Foundation.

## Background

Taeniasis and (neuro)cysticercosis are infections caused by the cestode *Taenia solium* (TS), involving a complex transmission cycle between the intermediate pig host and the definitive (also accidental intermediate) human host. When humans act as the accidental intermediate host, localization of larval-stage cysticerci in the central nervous system can result in neurocysticercosis (NCC), the main condition contributing to TS-associated morbidity and mortality, including epileptic seizures/epilepsy. TS is endemic across Latin America, sub-Saharan Africa (SSA), and Asia, especially South and South East Asia, in settings with low hygiene conditions where open defecation practices prevail and/or sanitation systems are insufficient to prevent exposure of infective material in human faeces to pigs ^1^. Most recent estimates of disease burden indicate that TS resulted in 1.61 (95% uncertainty interval=1.05–2.23) million disability-adjusted life years (DALYs) globally in 2017 for NCC-associated morbidity and mortality ^2^. This likely represents a substantial underestimation based on the difficulties inherent to assessing NCC prevalence and the way that disability weights have been attributed to NCC, which need to consider not only epilepsy but, at a minimum, also headache and neuropsychiatric co-morbidities ^3– 5^. Recent reviews have suggested that TS may also be present in other regions, such as Eastern Europe ^6^. Large economic consequences do not only pertain to the human public health sector, but also to the animal sector, resulting from reduced market value and market distortion associated with pig infection in the food-value chain, which disproportionately impacts the poorest farmers and communities ^7– 12^.

A variety of intervention options are available to tackle TS transmission in endemic settings. In the pig host, vaccines are available including TSOL18, alongside anthelmintic treatment using oxfendazole ^13^. The TSOL18 ^14^ vaccine has been licenced and made commercially available in India since November 2016, with registration underway in Uganda, Tanzania, Kenya, Nepal, Philippines, Thailand, and Sri Lanka, while oxfendazole was registered in Morocco for treatment against porcine cysticercosis (PCC) in June 2013 (see WHO article on Paranthic ^TM^ and Cysvax ^TM^). Intervention options under consideration include treatment of human taeniasis carriers based on mass drug administration (MDA) or on targeted treatment with either praziquantel or niclosamide ^13^. These treatments, e.g. praziquantel, could potentially be integrated with other neglected tropical disease (NTD) programs ^15^, such as those for schistosomiasis and soil-transmitted helminthiases. However, possible adverse neurological outcomes associated with treatment of NCC cases, especially at higher doses, may restrict the utility of praziquantel ^16^. Further structural changes/interventions that generate broader positive externalities such as impacting TS transmission, include improved sanitation and pig husbandry practices; however, their wide-scale implementation will mostly depend on longer-term economic development ^17^. Health education, such as the computer-based educational tool ‘The Vicious Worm’ ^18^, provides a low-cost, locally-adaptable, and implementable intervention for both short- and longer-term impact. Studies demonstrate improved and sustained knowledge uptake in Tanzanian health- and agriculture-sector professionals ^19, 20^, as well as in rural Zambian primary-school children ^21^. In a rural Mexican community, health education, developed with community-participation, showed reductions in pig foraging behaviour and access to infective material, accompanied by reductions in pig cysticercosis prevalence ^22^. Improvement in knowledge areas associated with reducing risk factors through health education, directed at school-age children ^23^, has highlighted the role of health education campaigns. Other community-based participatory educational interventions in Burkina Faso have also demonstrated a marked reduction in human cysticercosis incidence and prevalence ^24^. A community-led total sanitation (CLTS) intervention in Zambia did not reduce porcine cysticercosis prevalence, with sanitation practices and cysticercosis awareness largely unchanging ^25^, further indicating the importance of knowledge uptake. A recent systematic review analysed the available evidence on the effectiveness of TS intervention options ^26^, concluding that combined human- and pig-focussed interventions are the most promising strategies for achieving rapid declines in infection ^27^ and enhancing prospects for regional elimination ^28^. This further supports the argument for a One Health approach, including humans and non-human animals as well as the environment. In addition, integrated knowledge translation, where knowledge-users work with researchers throughout the research process ^29^, as well as contextualized policy- and practice-transfer mechanisms seem important for sustainability of the obtained results. In 2012, the World Health Organization (WHO) NTD roadmap called for the establishment of a validated strategy for TS control and elimination by 2015, and for interventions to be scaled up in selected countries by 2020 ^30^. However, a validated strategy has not yet been established. There are currently TS pilot control programs under assessment in Madagascar, with pilots also planned for Vietnam and China ^31^. Currently, there are no specific TS programmatic goals as identified for other NTD programs, reflecting the relatively early stage of consolidation of an optimal disease control strategy compared to other NTDs.

A recent WHO consultation was held to gather evidence to support a new NTD roadmap for post-2020 targets and milestones. Transmission dynamics models can help address evidence gaps by assessing, *in silico*, the feasibility and potential impact of different intervention strategies to achieve proposed 2021–2030 targets. Dixon *et al.* ^32^ recently conducted a systematic review and analysis of available TS transmission models, identifying four mathematical/computational/statistical models (among them three transmission dynamics models), and a conceptual ‘logical’ framework ^33^. Models included a decision-tree ^34^, Reed-Frost ^35^, individual-based ^36^, and population-based ^37^ frameworks. (Other models have been published since this review ^38, 39^.) On-going development of cystiSim ^36^ and EPICYST ^37^ within a collaborative umbrella, has led to the establishment of ‘CystiTeam’, a partnership between the groups that developed these two models (based at the University of Copenhagen/Scientific Institute of Public Health, Brussels, and Imperial College London, respectively) and groups of epidemiologists, veterinarians, clinicians, one-health experts and program stakeholders. With the development of the new 2030 NTD goals in mind, Table 1 outlines the current NTD goals for TS (2015, 2020) and the proposed goals for 2030, with a summary of their technical feasibility, requirements, and most prominent knowledge gaps and associated risks.

**Table 1.** Summary of modelling insights and challenges for reaching the World Health Organization (WHO) 2030 goals for *Taenia solium* taeniasis/cysticercosis.

**Current WHO Goal:**	Validated strategy for control of *Taenia solium* (TS) taeniasis/cysticercosis available (2015) ^30^. Interventions scaled up in selected countries for *T. solium* taeniasis/cysticercosis control (2020) ^30^.
**2030 Target:**	Endemic countries with intensified control in hyperendemic areas.
**Is the new target technically** **feasible under the current** **disease strategy?**	Difficult to ascertain as the optimal combination of intervention strategies for intensified control has not yet been established/validated, specific programmatic goals have not been proposed, and the extent of hyperendemic areas in endemic countries has not yet been delineated. Modelling can inform the design and evaluation of pilot and large-scale control programs with current (and complementary) intervention strategies in settings of varying endemicity, as well as contribute to the identification of optimal combinations of interventions ^32– 39^ cystiSim and EPICYST applicable, with cystiSim already in use (Zambia/PAHO) ^36, 40^.
**What is required to achieve** **the target? (updated strategy,** **use of new tools, etc.)**	Standardised definition of programmatic goals for TS control put forward by WHO/expert group. Standardised monitoring protocols to evaluate progress of intervention strategies. Long-term intervention approaches to asses long-term epidemiological impact.
**Are current tools able to** **reliably measure the target?**	Substantial limitations with existing serological diagnostics (for the assessment of prevalence in humans and pigs), and broader access including neuroimaging facilities (for the assessment of disease burden). Necropsy in pigs most reliable measure of infection in the porcine population, but limitations remain in terms of assessing interventions’ long-term effectiveness (models can assist). Lack of treatment and management guidelines for taeniasis/(neuro)cysticercosis.
**What are the biggest** **unknowns?**	In which areas infection is present; true prevalence in humans and pigs (due to poor diagnostics/lack of necropsy data) within endemic areas. Adult tapeworm lifespan; impact of pig-to-people population ratio on transmission; processes regulating parasite acquisition in humans and pigs ^38^, influence of environment on egg dynamics. Health & economic burden ^7, 8^ and cost-effectiveness of interventions (DALYs likely to underestimate disease burden); possible use of the zDALYs metric ^41^. Linking infection to disease models, particularly to human neurocysticercosis (NCC) and epilepsy ^32^.
**What are the biggest risks?**	Long-term sustainability of interventions is uncertain.

*WHO: World Health Organization, TS: Taenia solium, PAHO: Pan American Health Organization, DALY: Disability-Adjusted Life Year, zDALY: Zoonotic Disability-Adjusted Life Year, NCC: neurocysticercosis.*

## Insights gained from TS transmission dynamics modelling

**Figure 1.**
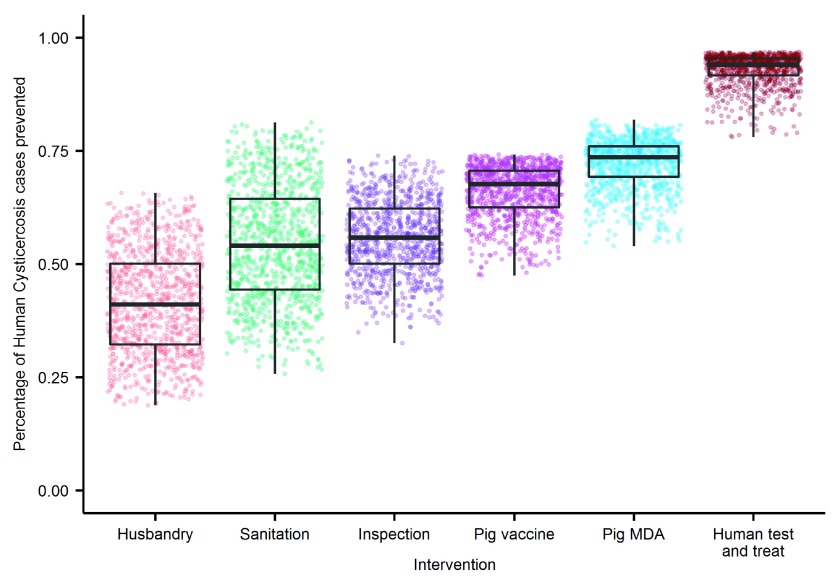
Impact of single interventions on the number of human cysticercosis cases. Box and whiskers represent the range of impact estimates from 1000 sensitivity draws of intervention efficacy parameters, the midline represents the median impact (on the proportion of Human Cysticercosis cases prevented), the hinges the 25 ^th^ and 75 ^th^ percentiles, and the whiskers the range. Points show individual run outputs. Due to the large amount of uncertainty in parameters estimates, the impact of parameter estimates was explored separately (see Figure 4 in Winskill *et al.* ^37^). This figure has been reproduced under the Creative Commons Attribution 4.0 International License (CC BY 4.0) from Winskill *et al.* ^37^.

**Figure 2.**
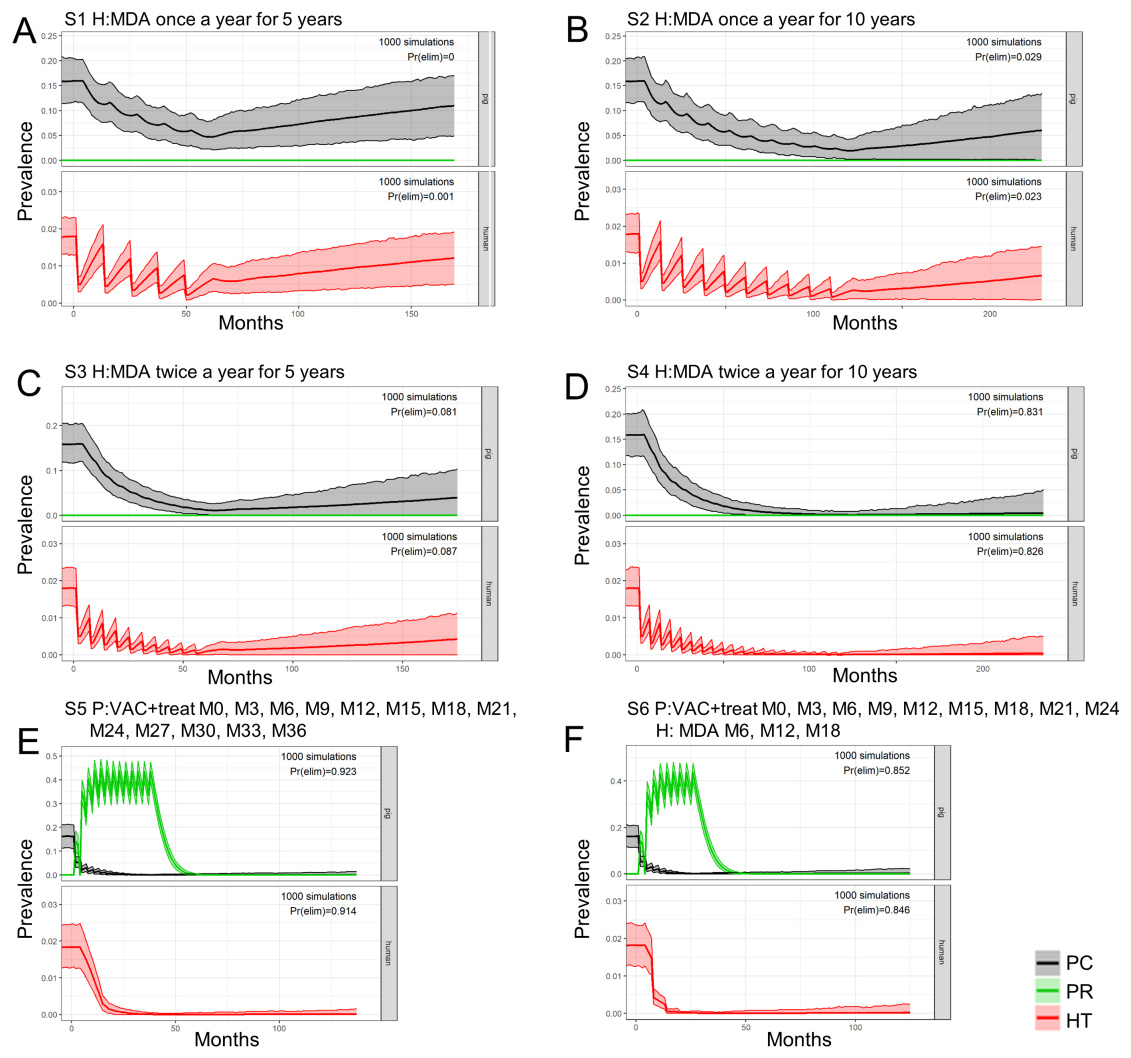
Simulation of various control scenarios for *Taenia solium* using cystiSim. The effectiveness of repeated mass drug administration (MDA) in humans (H) for taeniasis is compared with three-monthly interventions in pigs (P) involving vaccination and treatment, or a combination of pig interventions with strategic MDA in the human population after 1000 simulations (S). The impacts are shown on porcine cysticercosis (PC), immunity to *T. solium* infection in the pig population (PR), and human taeniasis (HT), with prevalence shown here as a proportion. The colored areas delineate the 95% uncertainty intervals for prevalence (proportion). Pr(elim) indicates the predicted probability of elimination of transmission (EOT) in the given scenario. Four scenarios are simulated which involve: MDA only in humans ( **A**– **D**): annual treatments for 5 or 10 consecutive years ( **A** and **B**, respectively), and biannual MDA for 5 or 10 consecutive years ( **C** and **D**, respectively). Two scenarios involve pig-focussed interventions. Each included three-monthly vaccination and oxfendazole treatment of the pig population. The first involves: vaccination and treatment in pigs ( **E**) for 3 years. The second ( **F**) also involves pig interventions, but includes three human MDA rounds, at 6, 12, and 18 months after the initiation of the interventions in pigs, over a total period of 2 years. This figure has been reproduced with permission from Braae *et al.* ^42^.

A quantitative comparison of TS transmission dynamics models has not yet been performed, and therefore it is not possible to formally compare the (cystiSim ^36^ and EPICYST ^37^) models at this stage (an aim of the CystiTeam partnership). The lack of standardised programmatic targets also restricts the ability to determine the effectiveness of different interventions with the available modelling frameworks. Current models, however, do shed some light on the potential impact of interventions under more generalised, illustrative scenarios. The population-based, deterministic, transmission model EPICYST ^37^ has highlighted, based on simulating a generic SSA setting, the higher efficacy (measured as the percentage of human cysticercosis cases prevented) of biomedical interventions (human test-and-treat, pig MDA and pig vaccination) compared to structural change-based interventions (improved husbandry, sanitation and meat inspection), although insufficient data and knowledge currently exist to parameterize accurately the latter ( Figure 1). In EPICYST ^37^, further developments are on-going to reflect age-specific stratification of interventions such as pig vaccination scheduling and to test the impact of praziquantel MDA in school-age children (SAC). cystiSim ^36^, an agent-based, stochastic, model is able to simulate age-structured, field-realistic interventions as well as bespoke treatment efficacy for varying settings. cystiSim has identified, within the context of an endemic district in Tanzania, that two-host interventions (human MDA plus pig vaccination and treatment), are optimal strategies to achieve elimination of transmission (EOT) if high coverage can be reached and sustained for prolonged periods ^36^. Both EPICYST ^37^ and cystiSim ^36^ agree that pig- and human-directed interventions are sensitive to coverage levels. Biomedical interventions were identified as more robust to changes in both coverage and efficacy compared to structural change-based interventions in EPICYST ^37^, although modelling of the latter requires collection of robust data. cystiSim ^36^ showed that coverage is particularly important when interventions target a single host, but the addition of an intervention targeting the second host can compensate for lower coverages ^36^. More recently, Braae *et al.* ^42^ used cystiSim ^36^ to explore intervention simulations deemed to be closely aligned to the (assumed) population biology of the parasite; for example, testing a combined pig intervention (TSOL18 vaccine and oxfendazole MDA) for a duration of 3 years to reflect the modelled average lifespan of the adult tapeworm ^43^. The EOT probability was >90% in this scenario (coverage: 75%; frequency: 3-monthly; duration: 3 years), suggesting a role for pig-only strategies if these can be implemented at high coverage and frequency for sufficiently long. This modelling study also indicated that the program duration could be reduced to 2 years with a similar EOT probability (>85%) with addition of human MDA after the first year (coverage: 80%; frequency: 6 monthly; no. of treatment rounds: 3; duration: 2 years). Inclusion of pig-focussed interventions (with or without human MDA) was substantially more effective than human MDA-only strategies (coverage: 80%; frequency: annual or 6-monthly; duration: 5 or 10 years) ( Figure 2).

## Use of TS transmission models to support intervention trials and programs

**Figure 3.**
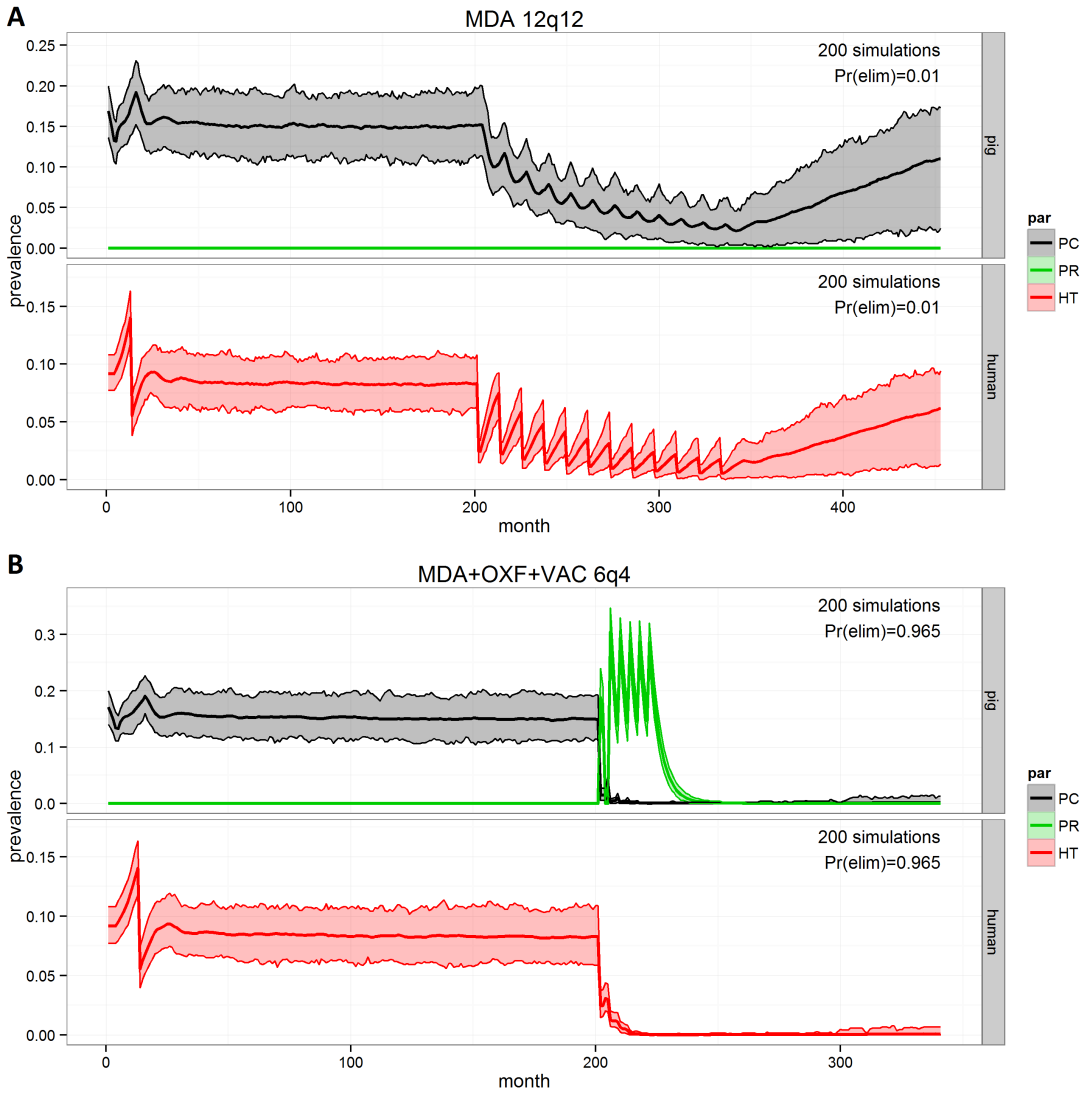
cystiSim output for different intervention scenarios aiming for control and elimination of *Taenia solium* in Zambia. Interventions aiming for control ( **A**) and elimination ( **B**): Pr _(elim)_: probability of elimination of transmission (EOT) of porcine cysticercosis (PCC) and human taeniasis (HT), MDA: human mass drug administration (praziquantel), OXF: porcine mass drug administration (oxfendazole), VAC: porcine vaccination (TSOL18), XqY: total number of X iterations of the intervention given at intervals of Y months. Interventions are introduced after 200 months. This figure has been reproduced with permission from Gabriël *et al.* ^40^. Y-axis shows prevalence as a proportion.

cystiSim has been used to inform potential control activities under the Pan American Health Organization (PAHO) and to support intervention design (intervention selection, coverage, frequency, duration) and assessment of options under consideration in the community-based intervention pilot project ‘CYSTISTOP’ in Zambia. CYSTISTOP commenced in 2015 and is due to end in 2020 ^44^. Modelling comparisons examine different intervention strategies, including mass or targeted treatment programs assumed to be feasible in ‘lower input/investment’ systems versus more intensive elimination strategies which focus on combined, higher-frequency interventions ^40^. Yearly pig oxfendazole MDA strategies (drug efficacy: 100%; therapeutic coverage: 90% of pigs aged ≥2 months; frequency: annual; no. rounds: 12; duration: 12 years) provided the most effective ‘control’ approach (EOT probability =75%), compared to human praziquantel MDA (drug efficacy: 95%; therapeutic coverage: 85% of humans aged ≥5 years; frequency: annual; no. rounds: 12; duration: 12 years), with EoT probability=1%. In terms of highly intensive elimination options, combined interventions (human and pig MDA with drug efficacy and coverage as above plus pig vaccination; frequency: 4-monthly; no. rounds: 6; duration: 2 years) was the most effective (EOT probability =96.5%) ( Figure 3). These simulated interventions will be evaluated using the final CYSTISTOP results for model validation.

## Considerations and barriers towards 2030 TS goals: the role of modelling

### Measuring the target

1) Timelines and feasibility of control goals.

Currently, a lack of internationally agreed goals (elimination as a public health problem, or elimination of transmission targets) curtail the identification of a validated TS control/elimination strategy. Closely linked to identifying specific goals is a clarification of the suitability of different strategies according to epidemiological setting, and the timeframes available to implement these strategies. Longer-term, lower input/investment ‘control’ options, implemented over many years are potentially more attractive in severely resource-constrained settings ^40^; however, these will require long-term political and financial support. Similar epidemiological outcomes could potentially be achieved under more intensive, shorter-duration interventions ^42^. Therefore, classifying feasible timeframes to achieve national and regional control goals ^45^, aligned not only to epidemiological/ecological settings, but also to realistic operational, logistic, economic and political conditions, will be an important precursor to testing and identifying setting-specific strategies. With the proposed overarching target of achieving “intensified control in hyperendemic settings”, technical definitions of endemicity levels are required, with subsequent mapping to identify hyperendemic areas within endemic countries. “Intensified control” will also need a technical definition; for example, achieving a proposed percentage prevalence or incidence reduction, or agreeing on what are the infection levels in humans and pigs that lead to a public/veterinary health problem. The level of intensified control will also need to be locally adaptable to conform to variable levels of resource constraints under realistic timeframes set within the 2020–2030 period. Collaboration between TS modelling groups and groups responsible for intervention trials, as demonstrated for CYSTISTOP in Zambia ^40^, can underpin model validation efforts, which would ultimately improve the predictive ability of TS transmission models. This will be an important step for projecting the impact of interventions under realistic timeframes, and therefore for identifying validated strategies, in a wider range of endemic settings.

2) Measures of effectiveness, diagnostic applications and limitations.

cystiSim, which has been used to simulate interventions in Tanzania ^36, 42^ and Zambia ^40^, models effectiveness in terms of the probability of achieving (local) elimination. Agreement needs to be reached on the validity of measures of effectiveness in field settings given the significant limitations of currently available diagnostic tools. Current serological methods are suboptimal for the diagnosis of PCC, lacking both specificity in areas where other *Taenia* species exist ^46, 47^ and sensitivity, particularly to light infections ^48^, restricting their use in near-elimination settings. Necropsy, consisting of full-carcass dissection for presence (and enumeration of cysterci), is the gold standard diagnostic for porcine PCC. However, necropsy is not suitable as a routine monitoring and evaluation (M&E) tool for control programs given the large number of animals required to detect a statistically meaningful impact on transmission. Such large necropsy sample sizes, removed from the general pig population, would (artefactually) influence transmission and local food-value chains ^45^. Different diagnostic methods may be more appropriate depending on transmission setting and stage of control. For epidemiological mapping of high-risk settings, pig tongue inspection may be suitable for identifying heavily infected animals and could play a wider role as a potential tool for rapid epidemiological assessment ^49^. As interventions are established in settings with moderate to high transmission, pigs of minimum slaughter age or weight can be initially screened, with a further sub-sample necropsied ^45, 50^, as implemented in the elimination trial in Northern Peru ^28^. Equally, limitations exist for human taeniasis diagnostics, including microscopy, coprology and antibody serology technology, with specificity issues in particular proving problematic for M&E purposes in typical endemic settings, where endemic taeniasis prevalence does not exceed 2% ^50^. Therefore, a crucial challenge for determining the effectiveness of intervention programs is defining what is meant by success and how proposed targets can be measured using available (or novel) diagnostic tools. Simulating the impact on transmission of control programs using mathematical models can help to assess whether “intensified control” could firstly be achieved with transmission dynamics in humans and pigs as observable in the field (adjusted by diagnostic characteristics), and, secondly, what would be the prospects for local true elimination were this possible in a specific location.

### Developing tailored, setting-specific strategies

1) Local practices.

Socio-cultural practices influence the TS transmission system, and as such, highlight requirements for tailored setting-specific intervention programs. One key area relates to the age/weight at which pigs are slaughtered and consumed, which varies dramatically and is suggested to heavily influence the effectiveness of pig-directed interventions ^33^. Cultural, religious and farming practices may also impact the timing of pork consumption ^33, 51^, and, therefore, improving knowledge of these practices and how they vary geographically could be used to construct a ‘pork consumption calendar’ ^33^. This, in turn, could be used to inform appropriate interventions, particularly regarding the timing of pig-directed interventions prior to peak pork-consumption periods. Health education will also be setting-dependent given varying husbandry and sanitation practices, and it would be highly valuable to test its impact with the transmission dynamics models as and when data become available on the impact of health education on TS transmission.

2) Spatial elements.

Spatial heterogeneity in the epidemiology of TS has been identified both at very small local scales and at higher spatial resolutions. Particularly in South American communities, local clustering of PCC ^52^ around human taeniasis carriers is a feature ^53– 56^ which needs more investigation in other locations. Local movement of pigs along value chains and human migratory patterns influence transmission at different scales and impact the prospects of elimination and resurgence ^34^. TS transmission models featuring spatial structuring could evaluate the impact of spatially heterogeneous transmission (at a variety of scales) on intervention effectiveness. At the highest spatial resolutions, a detailed overview of national/global distribution of TS is also lacking. CystiTeam members are currently working on models to inform on the global distribution of TS with the aim to map out areas in need of intervention and more accurately estimate the global burden of disease due to TS.

### Sustainability of intervention strategies

1) Integration with other NTD programs.

The cross-utility of anthelmintic drugs to target multiple helminth species presents opportunities for integration of NTD control programs. Co-distribution of schistosomiasis and TS has been identified in 17 African countries ^57^, while the presence of national-scale schistosomiasis control programs in more than 30 African countries presents opportunities for co-treatment of both helminths with praziquantel, albeit with risks of serious adverse events associated with NCC cases ^16^. Over a 4-year period, the impact of the National Schistosomiasis Control Programme in two endemic districts of Tanzania, targeting SAC, alongside a taeniasis “track-and-treat” intervention suggested a wider spillover impact on adult human taeniasis carriers and PCC prevalence ^15^. cystiSim simulated a school-based MDA for one of these districts, indicating that there was little impact on PCC prevalence and minimal impact on human taeniasis ^36^. These results indicate that a TS intervention constrained to targeting SAC (within an integrated school-based NTD program) would have limited impact on TS transmission, and will require additional TS-specific interventions to be effective. Reviewing taeniasis age-prevalence profiles and fitting Force-of-Infection (FOI), catalytic models may help elucidate whether age-targeted approaches are more effective in other epidemiological settings ^32^.

2) Cost implications and impact assessments.

Determining the most cost-effective interventions for TS requires further investigation. The ability to determine the relative cost-effectiveness compared to other NTD interventions will help to formulate an economic case for inclusion of TS control activities in national disease-control policies. Classical measures of cost-effectiveness, such as cost per DALY averted for different intervention options will be difficult to assess, as studies on longer-term impact on human cysticercosis incidence ^42^ and occurrence of NCC-related morbidity are difficult to implement. TS burden of disease and cost-impact studies have been conducted, focussing just on NCC human health impact in Mexico ^58^ and India ^59^, and more comprehensive assessments capturing costs in both human health and agricultural sectors in Tanzania ^7^, Mozambique ^8^, Cameroon ^60^, and South Africa ^11^. More recently, combined burden assessments using a zoonotic DALY framework (zDALY) ^41^ have been devised and implemented in Cameroon, and used to determine the cost-effectiveness of TS interventions in Lao People's Democratic Republic ^61^. TS transmission dynamics models provide added benefit by enabling dynamic (rather than static) burden of disease assessments. A key challenge here is linking human cysticercosis infection to morbidity because of the varying proportions of individuals developing morbidity, the time between exposure and disease onset, the highly pleomorphic nature of clinical NCC and the lack of neuroimaging facilities in resource-constrained settings to enable data collection alongside improved diagnostics ^32^. In the short-term, it is likely that designing cost-effectiveness studies on outcomes related to changes in PCC prevalence and human taeniasis indicators will be more feasible, but this will probably require superior diagnostic tools compared to those currently available.

## Future directions

**Figure 4.**
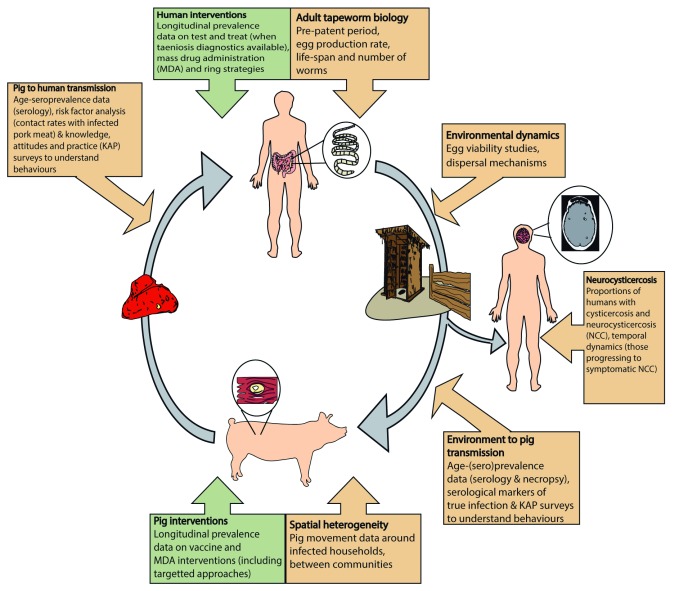
Lifecycle of *Taenia solium*. The lifecycle indicates, at each stage, key research gaps and data needs important for epidemiological modelling. This figure has been reproduced under the Creative Commons Attribution 4.0 International Licence (CC BY 4.0) from Dixon *et al*. ^32^. NCC: neurocysticercosis.

Addressing existing models’ structural and parametric uncertainty is a critical step towards enabling such models to increase their predictive capacity to assess the 2030 TS targets and provide robust support. There are several frameworks available ^32– 39^ with groups in CystiTeam working on improving the cystiSim and EPICYST models collaboratively. Knowledge gaps have been highlighted to further model development, including age-specific infection trends and local practices to inform setting-specific parameterization. Other biological parameters requiring further investigation include the average and distribution of the adult tapeworm lifespan, processes regulating parasite acquisition in humans and pigs, and exposure heterogeneity, which may manifest as aggregated (overdispersed) infection distributions at the population level (as attested by the distribution of cysticerci in pig populations ^49, 62, 63^). Identifying how current demographic assumptions impact transmission, such as the pig-to-people population ratio, need to be systematically tested between current TS transmission models. Figure 4 presents key research gaps and data needs to move the field forward.

## Data availability

### Underlying data

No data are associated with this article.
