# Supplementary material for: The World Health Organization 2030 goals for Taenia solium: Insights and perspectives from transmission dynamics modelling: CystiTeam Group for Epidemiology and Modelling of Taenia solium Taeniasis/Cysticercosis
Source: Gates Open Res. 2019 Dec 13;3:1546. Originally published 2019 Sep 26. [Version 2] doi: 10.12688/gatesopenres.13068.2 (PMC6820453; doi:10.12688/gatesopenres.13068.2)
Supplement: Peer review history

# Peer review: Reviewer response for version 1

**DOI:** 10.21956/gatesopenres.14200.r27945

**Published:** 2019-10-21

**Authors:** Kabemba Evans Mwape

**Affiliations:** 1 School of Veterinary Medicine, University of Zambia, Lusaka, Zambia

This paper is well written and very interesting to read as it brings to the fore what could probably be a huge milestone towards the control of *Taenia solium*, mathematical modelling of the transmission dynamics of the parasite. A robust transmission model is urgently needed in order to achieve the 2030 goals with regards *T. solium* control. It is encouraging that the cystiSim and EPICYST groups have teamed up to form CystiTeam. It is hoped that together with epidemiologists, veterinarians, clinicians, one health experts and program stakeholders, a model that clearly shows the effects of the various control on the parasite will be developed.

 Figure 2 clearly shows whether elimination of transmission can be achieved with the given control options including the frequency of implementation. My comment on this figure is to request that figure 2 E and F be enlarged to be the same size as the other figures, they seem smaller than A, B, C, D, E.

 Lastly, I would like to urge the authors to recheck the references as some of them do not tally e.g. reference number 31 should be changed to  34, the number in the text does not tally with the one on the list of references.

I confirm that I have read this submission and believe that I have an appropriate level of expertise to confirm that it is of an acceptable scientific standard.

# Response

**Published:** 2019-12-13

**Authors:** Maria-Gloria Basanez

**Affiliations:** Imperial College London, UK

**Reviewer #3 **(Kabemba Evans Mwape)

**Reviewer’s summary.** This paper is well written and very interesting to read as it brings to the fore what could probably be a huge milestone towards the control of *Taenia solium*, mathematical modelling of the transmission dynamics of the parasite. A robust transmission model is urgently needed in order to achieve the 2030 goals with regards *T. solium* control. It is encouraging that the cystiSim and EPICYST groups have teamed up to form CystiTeam. It is hoped that together with epidemiologists, veterinarians, clinicians, one health experts and program stakeholders, a model that clearly shows the effects of the various control on the parasite will be developed.

**Authors’ response. **We thank the reviewer for these positive and encouraging comments, and agree that this collaboration is an important step towards developing robust models to investigate control options.

**Reviewer’s comment 3.1. **Figure 2 clearly shows whether elimination of transmission can be achieved with the given control options including the frequency of implementation. My comment on this figure is to request that figure 2 E and F be enlarged to be the same size as the other figures, they seem smaller than A, B, C, D, E.

**Authors’ response. 3.1. **We thank the reviewer for these suggestions. However we are unable to edit the original files as the figures were taken from previous publications.

**Reviewer’s comment 3.2. **Lastly, I would like to urge the authors to recheck the references as some of them do not tally e.g. reference number 31 should be changed to 34, the number in the text does not tally with the one on the list of references.

**Authors’ response. 3.2. **We thank the reviewer for highlighting the referencing discrepancies (see also response to 2.2); these are corrected in the revised manuscript.

# Peer review: Reviewer response for version 1

**DOI:** 10.21956/gatesopenres.14200.r27943

**Published:** 2019-10-15

**Authors:** Joaquin M. Prada

**Affiliations:** 1 School of Veterinary Medicine, Faculty of Health and Medical Sciences, University Of Surrey, Guildford, UK

This paper summarizes the opinions of a group of mathematical modellers, veterinarians and clinical experts regarding the WHO 2030 goals for the control of cysticercosis. The manuscript is clear and well written and is illustrated by the outputs, from recent publications, of the mathematical models that the authors have developed.

 My only comment is clarification on the figures' units for the y-axis (I do take the point that these figures have been extracted as they were from previous work). Figure 1 label states percentage, but the axis is from 0 to 1. Figures 2 and 3 just state “prevalence” in the y-axis (prevalence is often showed as a percentage, but the axis might suggest it is being shown as a proportion?). I would recommend either changing the axis to be between 0 to 100%, or clarify in the labels which ones are proportions and/or percentages.

 The references in the figure labels also need to be corrected. Figure 1 references Winskill *et al.* as ref 23, but I believe it is ref 34. Similarly in Figures 2 & 3: Brae *et al.* Ref 31 and Gabriël *et al.* ref 25, when I believe those are ref 33 and ref 37 respectively. I would suggest re-checking all the references throughout to make sure they are correct.

I confirm that I have read this submission and believe that I have an appropriate level of expertise to confirm that it is of an acceptable scientific standard.

# Response

**Published:** 2019-12-13

**Authors:** Maria-Gloria Basanez

**Affiliations:** Imperial College London, UK

**Reviewer #2 **(Joaquin M. Prada)

**Reviewer’s summary.** This paper summarizes the opinions of a group of mathematical modellers, veterinarians and clinical experts regarding the WHO 2030 goals for the control of cysticercosis. The manuscript is clear and well written and is illustrated by the outputs, from recent publications, of the mathematical models that the authors have developed.

**Authors’ response to summary. **We thank the reviewer for these comments.

**Reviewer’s comment 2.1. **My only comment is clarification on the figures' units for the y-axis (I do take the point that these figures have been extracted as they were from previous work). Figure 1 label states percentage, but the axis is from 0 to 1. Figures 2 and 3 just state “prevalence” in the y-axis (prevalence is often showed as a percentage, but the axis might suggest it is being shown as a proportion?). I would recommend either changing the axis to be between 0 to 100%, or clarify in the labels which ones are proportions and/or percentages.

**Authors’ response. 2.1. **We thank the reviewer for these comments, and acknowledge the points raised; however, as the reviewer notes, these figures are extracted from previous publications and therefore difficult to modify. We have, as suggested, provided clarification in the figure legends as follows:

*“* ***Figure 1.*** ***Impact of single interventions on the number of human cysticercosis cases*** *. Box and whiskers represent the range of impact estimates from 1000 sensitivity draws of intervention efficacy parameters, the midline represents the median impact (on proportion of Human Cysticercosis cases prevented), the hinges the 25 ^th^ and 75 ^th^ percentiles, and the whiskers the range. Points show individual run outputs. Due to the large amount of uncertainty in parameters estimates, the impact of parameter estimates was explored separately (see Fig. 4* *in Winskill et al. ^37^* *). This figure has been reproduced under the Creative Commons Attribution 4.0 International License (CC BY 4.0) from Winskill et al. [37].”*

            

*“* ***Figure 2.*** ***Simulation of various control scenarios for Taenia solium using cystiSim.*** *The effectiveness of repeated mass drug administration (MDA) in humans (H) for taeniasis is compared with three-monthly interventions in pigs (P) involving vaccination and treatment, or a combination of pig interventions with strategic MDA in the human population after 1000 simulations (S). The impacts are shown on porcine cysticercosis (PC), immunity to T. solium infection in the pig population (PR), and human taeniasis (HT), with prevalence shown here as a proportion. The coloured areas delineate the 95% uncertainty intervals for prevalence (proportion). Pr(elim) indicates the predicted probability of elimination of transmission (EOT) in the given scenario. Four scenarios are simulated which involve: MDA only in humans (A–D): annual treatments for 5 or 10 consecutive years (A and B, respectively), and biannual MDA for 5 or 10 consecutive years (C and D, respectively). Two scenarios involve pig-focussed interventions. Each included three-monthly vaccination and oxfendazole treatment of the pig population. The first involves: vaccination and treatment in pigs (E) for 3 years. The second (F) also involves pig interventions, but includes three human MDA rounds, at 6, 12, and 18 months after the initiation of the interventions in pigs, over a total period of 2 years. This figure has been reproduced with permission from Braae et al. [36].*

 “ ***Figure 3.*** ***cystiSim output for different intervention scenarios aiming for control and elimination of Taenia solium in Zambia.*** *Interventions aiming for control (A) and elimination (B): Pr _(elim)_: probability of elimination of transmission (EOT) of porcine cysticercosis (PCC) and human taeniasis (HT), MDA: human mass drug administration (praziquantel), OXF: porcine mass drug administration (oxfendazole), VAC: porcine vaccination (TSOL18), XqY: total number of X iterations of the intervention given at intervals of Y months. Interventions are introduced after 200 months. This figure has been reproduced with permission from Gabriel et al. [43]. Y-axis shows prevalence as a proportion.”*

**Reviewer’s comment 2.2. **The references in the figure labels also need to be corrected. Figure 1 references Winskill *et al.* as ref 23, but I believe it is ref 34. Similarly in Figures 2 & 3: Brae *et al.* Ref 31 and Gabriël *et al.* ref 25, when I believe those are ref 33 and ref 37 respectively. I would suggest re-checking all the references throughout to make sure they are correct.

**Authors’ response. 2.2. **We thank the reviewer for highlighting these discrepancies. We have checked and modified the reference numbers in the revised manuscript also accounting for the fact that other reviewers have suggested inclusion of additional references.

# Peer review: Reviewer response for version 1

**DOI:** 10.21956/gatesopenres.14200.r27944

**Published:** 2019-10-01

**Authors:** Ana Flisser Steinbruch

**Affiliations:** 1 National Autonomous University of Mexico, Mexico City, Mexico

This paper is very interesting and well written. I have only a few comments:

 Page 1, right column, please delete as marked: This further supports the argument for a One Health approach, including humans and non-human animals as well as the environment.

 The only mention to health education is exactly before 2) Spatial elements, as follows "Health education will also be setting-dependent given varying husbandry and sanitation practices, and it would be highly valuable to test its impact with the transmission dynamics models as and when data become available on the impact of health education on TS transmission". To this regard, there are a few studies available in which health education was evaluated (Mexico and Tanzania) but no mention is given to these studies.

 In spatial elements, there are three references regarding clustering of pig cysticercosis to human taeniasis, but the original reference is absent, could the authors please include it: Sarti-Gutierrez *et al. *(1988) ^1^. Furthermore, in 1) Integration with other NTD programs, the authors state that there are no publications on the "risks of serious adverse events associated with NCC cases [ASW, unpublished data]. I recommend the authors to read Flisser *et al. *(1993) ^2^. 

 In Figure 4, more or less where red pork meat is seen, I would add another window of information regarding KAP (knowledge, attitudes and practices) needed to be able to control cysticercosis/taeniasis

 I especially enjoyed Table 1.

I confirm that I have read this submission and believe that I have an appropriate level of expertise to confirm that it is of an acceptable scientific standard.

## References

[1] : Taenia solium taeniasis and cysticercosis in a Mexican village. *Trop Med Parasitol*.1988;39(3) :194-8 3194663

[2] : Neurological symptoms in occult neurocysticercosis after single taeniacidal dose of praziquantel. *Lancet*.1993;342(8873) : 10.1016/0140-6736(93)91743-6 748 10.1016/0140-6736(93)91743-6 8103859

# Response

**Published:** 2019-12-13

**Authors:** Maria-Gloria Basanez

**Affiliations:** Imperial College London, UK

**Reviewer #1 **(Ana Flisser Steinbruch)

**Reviewer’s summary.** This paper is very interesting and well written.

**Authors’ response to summary. **We thank the reviewer for these comments.

**Reviewer’s comment 1.1. **Page 1, right column, please delete as marked: This further supports the argument for a One Health approach, including humans and non-human animals as well as the environment.

**Authors’ response 1.1. **We thank the Reviewer for this comment; however, we argue that this statement should be maintained in the manuscript, with the previous sentence supporting that combined interventions (aimed at both human and pig hosts) advocate for a cross-sector, One Health strategy, to control *T. solium*:

*“A recent systematic review analysed the available evidence on the effectiveness of TS intervention options ^26^, concluding that combined human- and pig-focussed interventions are the most promising strategies for achieving rapid declines in infection ^27^ and enhancing prospects for regional elimination ^27^.”*

**Reviewer’s comment 1.2. **The only mention to health education is exactly before 2) Spatial elements, as follows "Health education will also be setting-dependent given varying husbandry and sanitation practices, and it would be highly valuable to test its impact with the transmission dynamics models as and when data become available on the impact of health education on TS transmission". To this regard, there are a few studies available in which health education was evaluated (Mexico and Tanzania) but no mention is given to these studies.

**Authors’ response 1.2. **We appreciate the reviewer’s insight into this area of *T. solium* research, and her suggestion that other references may be used. We have, therefore, included the following text in the revised manuscript:

*“In a rural Mexican community, health education, developed with community-participation, showed reductions in pig foraging behaviour and access to infective material, accompanied by reductions in pig cysticercosis prevalence ^22^. Improvement in knowledge areas associated with reducing risk factors through health education, directed at school-age children ^23^, has highlighted the role of health education campaigns.”*

 With the additional references:

 22. Sarti E, Flisser A, Schantz PM, Gleizer M, Loya M, Plancarte A, *et al*.: **Development and evaluation of a health education intervention against *Taenia solium* in a rural community in Mexico.** *Am J Trop Med Hyg.* 1997; 56(2): 127-132.

 23. Mwidunda SA, Carabin H, Matuja WBM, Winkler AS, Ngowi HA. **A school based cluster randomised health education intervention trial for improving knowledge and attitudes related to *Taenia solium* cysticercosis and taeniasis in Mbulu District, Northern Tanzania**. *PLoS One. *2015; 10(2): e0118541.

**Reviewer’s comment 1.3. **In spatial elements, there are three references regarding clustering of pig cysticercosis to human taeniasis, but the original reference is absent, could the authors please include it: Sarti-Gutierrez et al. (1988). Furthermore, in 1) Integration with other NTD programs, the authors state that there are no publications on the "risks of serious adverse events associated with NCC cases [ASW, unpublished data]. I recommend the authors to read Flisser et al. (1993).

**Authors’ response 1.3. **We thank the reviewer for highlighting an original reference on the clustering issue which we have now included:

 53. Sarti-Gutierrez EJ, Schantz PM, Lara-Aguilera R, Gomez Dandoy H, Flisser A. ***Taenia solium* taeniasis and cysticercosis in a Mexican village.** *Trop Med Parasitol*. 1988; 39(3): 194-98.

 We have also included the suggested reference regarding risks of serious adverse events associated with treatment of NCC cases:

 16. Flisser A, Madrazo I, Plancarte A, Schantz P, Allan J, Craig P, Sarti E. **Neurological symptoms in occult neurocysticercosis after single taeniacidal dose of praziquantel**. *Lancet.* 1993; 342(8873): 748.

**Reviewer’s comment 1.4. **In Figure 4, more or less where red pork meat is seen, I would add another window of information regarding KAP (knowledge, attitudes and practices) needed to be able to control cysticercosis/taeniasis.

**Authors’ response 1.4. **We thank the reviewer for this suggestion, and have added KAP (knowledge, attitudes and practices) to the existing “Pig to human transmission” and “Environment to pig transmission” boxes.

**Reviewer’s comment 1.5. **I especially enjoyed Table 1.

**Authors’ response 1.5. **We thank the reviewer for this appreciative comment.
